# Common Genetic Variants Explain the Majority of the Correlation Between Height and Intelligence: The Generation Scotland Study

**DOI:** 10.1007/s10519-014-9644-z

**Published:** 2014-02-20

**Authors:** Riccardo E. Marioni, G. David Batty, Caroline Hayward, Shona M. Kerr, Archie Campbell, Lynne J. Hocking, Generation Scotland, David J. Porteous, Peter M. Visscher, Ian J. Deary

**Affiliations:** 1 Centre for Cognitive Ageing and Cognitive Epidemiology, University of Edinburgh, 7 George Square, Edinburgh, EH8 9JZ UK; 2 Medical Genetics Section, Centre for Genomics and Experimental Medicine, Institute of Genetics and Molecular Medicine, University of Edinburgh, Edinburgh, EH4 2XU UK; 3 Queensland Brain Institute, The University of Queensland, Brisbane, QLD 4072 Australia; 4 Department of Epidemiology and Public Health, University College London, 1-19 Torrington Place, London, WC1E 6BT UK; 5 Alzheimer Scotland Dementia Research Centre, University of Edinburgh, 7 George Square, Edinburgh, EH8 9JZ UK; 6 Medical Research Council Human Genetics Unit, Institute of Genetics and Molecular Medicine, University of Edinburgh, Edinburgh, EH4 2XU UK; 7 Musculoskeletal Research Programme, Division of Applied Medicine, University of Aberdeen, Aberdeen, AB25 2ZD UK; 9 University of Queensland Diamantina Institute, The University of Queensland, Translational Research Institute, Brisbane, QLD 4072 Australia; 10 Department of Psychology, University of Edinburgh, Edinburgh, EH8 9JZ UK

**Keywords:** Height, Intelligence, Molecular genetics, Genetic correlation, Generation Scotland

## Abstract

Greater height and higher intelligence test scores are predictors of better health outcomes. Here, we used molecular (single-nucleotide polymorphism) data to estimate the genetic correlation between height and general intelligence (g) in 6,815 unrelated subjects (median age 57, IQR 49–63) from the Generation Scotland: Scottish Family Health Study cohort. The phenotypic correlation between height and g was 0.16 (SE 0.01). The genetic correlation between height and g was 0.28 (SE 0.09) with a bivariate heritability estimate of 0.71. Understanding the molecular basis of the correlation between height and intelligence may help explain any shared role in determining health outcomes. This study identified a modest genetic correlation between height and intelligence with the majority of the phenotypic correlation being explained by shared genetic influences.

**Electronic supplementary material:**

The online version of this article (doi:10.1007/s10519-014-9644-z) contains supplementary material, which is available to authorized users.

## Background

Evidence from observational studies suggests that better cognitive performance (as assessed by IQ-type tests) is associated with better health outcomes and lower mortality risk (Calvin et al. 2011; Deary et al. 2010; Whalley and Deary 2001). Greater height is also associated with a lower risk of a series of health outcomes including coronary heart disease, stroke, accidents and suicide (Batty et al. 2009; Lee et al. 2009; Paajanen et al. 2010; Whitley et al. 2010).

Height and intelligence are positively correlated (Gale 2005), with r typically between 0.10 and 0.20 (Keller et al. 2013). Both traits are partly heritable: behaviour genetic studies, mostly using twin samples, provide narrow sense heritability estimates of 70–90 % for height (Macgregor et al. 2006; Silventoinen et al. 2003) and 40–70 % for intelligence (Calvin et al. 2012; Haworth et al. 2010). More recently, molecular-based studies indicate that about 45 % of the variation in height (Yang et al. 2010) and 25–50 % of the variance in intelligence (Benyamin et al. 2013; Davies et al. 2011) can be explained by additive effects of common single nucleotide polymorphisms (SNPs). However, to date, genetic correlations between height and intelligence have been calculated using only twin or family based data, in which relatedness is defined via pedigree information. They indicate varying estimates of the height-intelligence genetic correlation that range between 0.08 and 0.30 (Beauchamp et al. 2011; Keller et al. 2013; Silventoinen et al. 2006; Sundet et al. 2005). Whereas the benefits of the twin approach include testing for pleiotropic and assortative mating contributions to the correlation (Keller et al. 2013), and the opportunity to incorporate rare variants and non-additive genetic variation, drawbacks include the equal environments assumption (that is, there is no differential environment for monozygotic and dizygotic twins) and an inability to target specific causal variants and molecular pathways.

Here, we used a molecular genetic approach to examine the genetic correlation between height and intelligence in a large sample of unrelated adults. The method applied investigates phenotypic similarities in genetically similar (based on molecular level SNP data) unrelated individuals.

## Methods

Generation Scotland: the Scottish Family Health Study (GS:SFHS) is a family structured, population-based cohort study (Smith et al. 2006, 2012). A full description of the study is provided elsewhere (Smith et al. 2006, 2012; www.generationscotland.org/). In brief, over 24,000 participants were recruited between 2006 and 2011. Probands (n = 7,953) were aged between 35 and 65 years and were registered with participating general medical practitioners (GPs) in the Glasgow, Tayside, Ayrshire, Arran, and North-East regions of Scotland. These individuals were not ascertained on the basis of having any particular disorder. Their family members were also recruited to yield the full study sample.

### Genotyping Sample

Genome-wide data were collected on a sub-sample of 10,000 participants using the Illumina HumanOmniExpressExome-8 v1.0 DNA Analysis BeadChip and Infinium chemistry (Gunderson 2009). Blood samples (or saliva from postal and a few clinical participants) from GS:SFHS participants were collected, processed and stored using standard operating procedures and managed through a laboratory information management system at the Wellcome Trust Clinical Research Facility Genetics Core, Edinburgh (Kerr et al. 2013). The yield of DNA was measured using picogreen and normalised to 50 ng/μl before genotyping. The arrays were imaged on an Illumina HiScan platform and genotypes were called automatically using GenomeStudio Analysis software v2011.1. After quality control, there were a total of 594,824 SNPs available for analysis on 9,863 individuals, which included family trios and quads in addition to unrelated participants. A genetic threshold of 0.025 (between second and third cousins) was used to remove potential shared environment effects (Yang et al. 2010, 2011). This left an unrelated sample size of 6,815. SNPs with a MAF below 1 % were excluded prior to the analysis.

### Ethics Statement

All components of GS:SFHS received ethical approval from the NHS Tayside Committee on Medical Research Ethics (REC Reference Number: 05/S1401/89). GS:SFHS has also been granted Research Tissue Bank status by the Tayside Committee on Medical Research Ethics (REC Reference Number: 10/S1402/20), providing generic ethical approval for a wide range of uses within medical research.

### Cognition and Height

General intelligence was assessed by extracting the first, unrotated principal component from four cognitive tests that measured processing speed (Wechsler digit symbol substitution task—DST; Wechsler 1998a), verbal declarative memory (Wechsler logical memory test—LM; sum of immediate and delayed recall of one paragraph; Wechsler 1998b), executive function (verbal fluency test—VFT; using the letters C, F, and L, each for 1 min; Lezak 1995), and vocabulary (the Mill Hill vocabulary scale—MHVS; junior and senior synonyms combined; Raven et al. 1977). This component, which we label g, explained 45 % of the variance of the four tests, each of which loaded strongly on the component (0.64–0.72). These loadings represent the weight that each individual’s (standardised) cognitive test scores needs to be multiplied by in order to obtain their g score.

Height was measured during clinical examination by asking each participant to remove their shoes and to stand (i) as erectly as possible with their back and shoulders against the freestanding measurement device, (ii) with heels together and feet angled at about 60°, and (iii) with head held in the Frankfort horizontal plane, where the inferior border of the bony orbit is in line with the groove at the top of the tragus of the ear. Height to the nearest half centimetre was then measured during quiet breathing, with the horizontal arm of the measuring unit being kept at a rigid right angle to the scale.

### Statistical Analyses

Age-, sex-, and population stratification-adjusted residuals for both g and height were computed by linear regression. The number of ancestry components was determined by comparing the log-likelihoods and residual errors from linear regression models of the traits on age, sex, and up to 20 principal components (Supplementary Fig. 1). Based on these results, we adjusted for 14 components, which accounted for 1.0 % of the variance in g, and 0.8 % of the variance in height.

The residual values were carried forward to the genome-wide complex trait analyses—GCTA (Yang et al. 2010, 2011). Initially, univariate models were run for each trait to investigate the proportion of phenotypic variance that is explained by common genetic variants and variants that are in high linkage disequilibrium with them. The univariate GCTA estimates for g have been reported previously (Marioni et al., in press). Bivariate GCTA models (Lee et al. 2012) were then run to obtain estimates of the genetic correlation and bivariate heritability between height and g.

## Results

Descriptive details of the genotyped Generation Scotland cohort and the analysis cohort of unrelated study members are presented in Table 1. The median age of the genotyped cohort was 54 years (IQR 43–62), and 59 % of participants were female. As anticipated, men (mean height 176 cm [SD 7]) were taller than women (mean height 162 cm [SD 7]). The age- and sex-adjusted phenotypic correlation between height and g was 0.16 (SE 0.01). The unrelated subjects are representative of the full genotyped cohort, with negligible differences in the summary information.

**Table 1:** Characteristics of the genotyped Generation Scotland cohort study members

Variable	Genotyped cohort			Unrelated genotyped cohort		
	n	Mean (SD)		n	Mean (SD)	
Demographics						
Age (years)	10,000	54^a^	43–62	6,815	57^a^	49–63
Sex—female	10,000	5,864^b^	59	6,815	4,002^b^	59
Height (cm) (n = 9,969)						
Female	5,843	162	7	3,987	162	6
Male	4,126	176	7	2,805	175	7
Cognitive						
Digit symbol test	9,862	70.2	17.2	6,718	68.4	16.8
Verbal fluency	9,883	40.5	12.0	6,736	41.0	12.2
Logical memory	9,880	30.7	8.0	6,731	30.3	7.9
Mill Hill vocabulary scale	9,824	30.8	4.6	6,694	31.2	4.7

^a^Median (quartiles)

^b^n (%)

The univariate and bivariate GCTA results are presented in Table 2, with full output in Supplementary Tables I and II. The proportion of variance in the traits that was explained by the common SNPs was 0.58 (SE 0.05) for height, and 0.28 (SE 0.05) for g.

**Table 2:** Age-, sex-, and population stratification-adjusted univariate and bivariate GCTA-derived estimates

Univariate GCTA estimates	n	est^a^	SE				
g	6,609	0.28	0.05				
Height	6,792	0.58	0.05				

Bivariate GCTA estimates	n	r_G_	SE	r_E_	biv h^2^	Pearson r	SE
g:Height	6,609:6,792	0.28	0.09	0.08	0.71	0.16	0.01

*g* general intelligence derived from principal components analysis, *r*_*G*_ genetic correlation, *r*_*E*_ environmental correlation, *biv h* ^*2*^ bivariate heritability, *Pearson r* Pearson phenotypic correlation

^a^The proportion of variance in the phenotype explained by common genetic variants

The genetic correlation for height and g was 0.28 (SE 0.09). Bivariate heritability estimates indicated that the majority (71 %) of the phenotypic correlation between height and g was explained by common additive genetic variants.

## Discussion

In this study we found a moderate and statistically significant genetic correlation between height and general intelligence, g. Whereas the phenotypic correlation between these measures was small (~0.16), the bulk of this correlation can be explained by common additive genetic variants, and variants in linkage disequilibrium with them.

The main limitation of our analysis compared to previously reported findings from twin and family based data (Beauchamp et al. 2011; Keller et al. 2013; Silventoinen et al. 2006; Sundet et al. 2005) is its inability to determine the degree to which the correlation depends on assortative mating versus pleiotropy (Keller et al. 2013). In their study, Keller et al. (2013) used a bivariate nuclear twin family study design; however, there is no obvious analogue to such an approach for molecular level data. A further limitation of the model is that it only considered common variants (MAF > 1 %) and additive effects. This does not bias the estimate of the genetic correlation (Trzaskowski et al. 2013) but does result in lower univariate estimates for the traits compared to the corresponding figures from twin studies, which include non-additive genetic effects and the influence of rare variants.

The primary advantage of our analysis is that we can quantify the common additive SNP contribution to the phenotypic correlation and the overlap of these SNP effects on the variance explained in each of the two traits. Such an approach determines the molecular signal size and is the start to understanding the underlying biology of the genetic correlation. Other strengths include the large sample size, which is essential for calculating precise genetic correlation estimates; the phenotypic measure of g, which was derived from a battery of four cognitive tests; and the ethnic homogeneity of the Generation Scotland sample.

The findings from our molecular genetic study are in accordance with those reported in previous twin and family based analyses, where estimates of the height-cognition genetic correlation vary between 0.20 and 0.35 (Beauchamp et al. 2011; Silventoinen et al. 2006; Sundet et al. 2005). However, the most recent study in this area presented more modest genetic correlations of 0.08 for men, and 0.17 for women in a population of around 8,000 subjects from nearly 3,000 families (Keller et al. 2013). Reasons for cross-cohort differences have been reported by Keller et al. (2013) and may include birth cohort effects that are related to nutritional differences, childhood infections, and social circumstances.

Understanding the genetic correlation between height and IQ is important in life-course research as both traits have been described as predictors of health outcomes and mortality. Short stature has been linked to cardiovascular disease risk (Paajanen et al. 2010), and numerous other health outcomes (Batty et al. 2009; Lee et al. 2009; Whitley et al. 2010). Higher IQ has been linked to lower mortality risk, in addition to a decreased risk of a host of health outcomes such as coronary heart disease, stroke, accidents, and suicide (Calvin et al. 2011; Deary et al. 2010; Whalley and Deary 2001). There is also a complex interplay between height and intelligence and their role in both development and late-life ageing. Cognitive change from age 11 to 79 years has been shown to predict height loss between ages 79 and 87 years (Starr et al. 2010). The reason for the intelligence-health relation is not fully understood. One of several non-exclusive possibilities is that height and intelligence are both markers of ‘system integrity’ (Deary 2012). The present phenotypic and genetic correlation between them provides partial support for that suggestion. Further research should examine whether their shared genetic variation is associated with health outcomes, and seek to identify the molecular mechanisms.

In conclusion, we found a moderate molecular genetic correlation between height and intelligence. Furthermore, the majority of the phenotypic correlation between the traits can be explained by shared genetic influences.

## Electronic supplementary material

Below is the link to the electronic supplementary material.

Supplementary material 1 (PDF 25 kb)

Supplementary material 2 (PDF 4 kb)
