# Supplementary material for: Common Genetic Variants Explain the Majority of the Correlation Between Height and Intelligence: The Generation Scotland Study
Source: Behav Genet. 2014 Feb 20;44(2):91–6. doi: 10.1007/s10519-014-9644-z (PMC3938855; doi:10.1007/s10519-014-9644-z)
Supplement: Supplementary file 1 — Supplementary material 1 (PDF 25 kb) [file 10519_2014_9644_MOESM1_ESM.pdf]

Supplementary Table I: Age-, sex-, and population stratification-adjusted univariate GCTA models for cognition and height.

	n	V(G)	SE	V(e)	SE	Vp	SE	V(G)/Vp	SE
g	6,609	0.269	0.052	0.685	0.051	0.954	0.017	0.282	0.054
Height	6,792	22.903	2.194	16.836	2.048	39.739	0.694	0.576	0.052

g: general intelligence derived from principal components analysis.

V(G): genetic variance, V(e): residual variance, Vp: phenotypic variance, V(G)/Vp: ratio of genetic variance to phenotypic variance.

Supplementary Table II: Age-, sex-, and population stratification-adjusted bivariate GCTA model of cognition and height.

	n	$V(G)_{tr1}$	SE	$V(G)_{tr2}$	SE	$C(G)_{tr12}$	SE	$V(e)_{tr1}$	SE	$V(e)_{tr2}$	SE	$C(e)_{tr12}$	SE	Vp_{tr1}	SE	Vp_{tr2}	SE	$V(G)/Vp_{tr1}$	SE	$V(G)/Vp_{tr2}$	SE	r_G	SE
g :	6,609:																						
Height	6,792	0.264	0.052	22.878	2.194	0.682	0.242	0.690	0.052	16.859	2.048	0.285	0.233	0.954	0.017	39.737	0.694	0.277	0.054	0.576	0.052	0.277	0.095

g: general intelligence derived from principal components analysis.

$V(G)_{tri}$: genetic variance for trait *i*, $V(e)_{tri}$: residual variance for trait *i*, Vp_{tri}: phenotypic variance for trait *i*, $V(G)/Vp_{tri}$: ratio of genetic variance to phenotypic variance for trait *i*, $C(G)_{tri}$: genetic covariance for trait *i*, $C(e)_{tri}$: residual covariance for trait *i*, r_G: genetic correlation.

Bivariate heritability was calculated as $C(G)_{tr12} / (C(G)_{tr12} + C(e)_{tr12})$

Supplementary Figures

Supplementary Figure 1: Comparison of log-Likelihoods and residual errors for linear regression models of age-, sex-, and population stratification (up to 20 principal components)-adjusted cognition and height.
