# Supplementary figures and images for: Common Genetic Variants Explain the Majority of the Correlation Between Height and Intelligence: The Generation Scotland Study
Source: Behav Genet. 2014 Feb 20;44(2):91–6. doi: 10.1007/s10519-014-9644-z (PMC3938855; doi:10.1007/s10519-014-9644-z)

**g**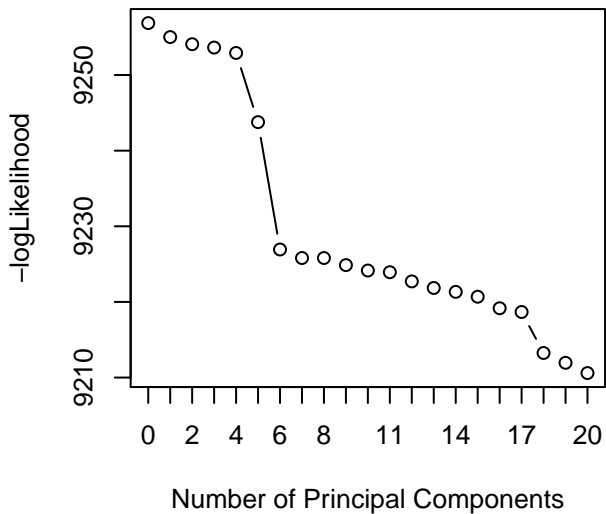**g**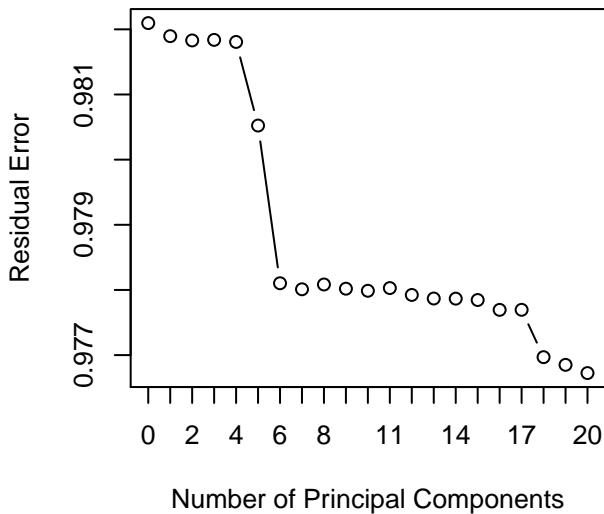**Height**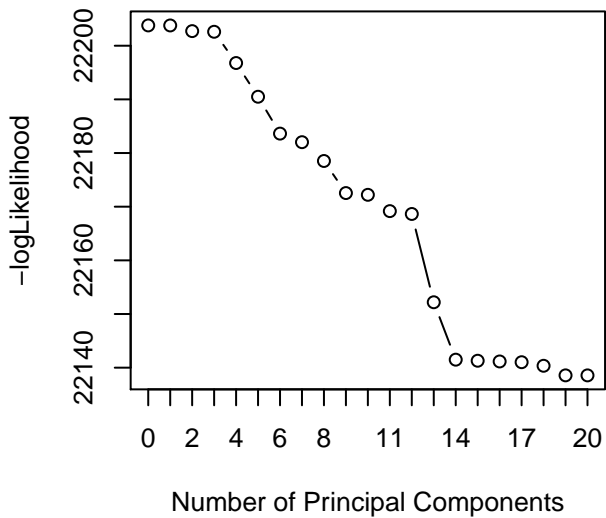**Height**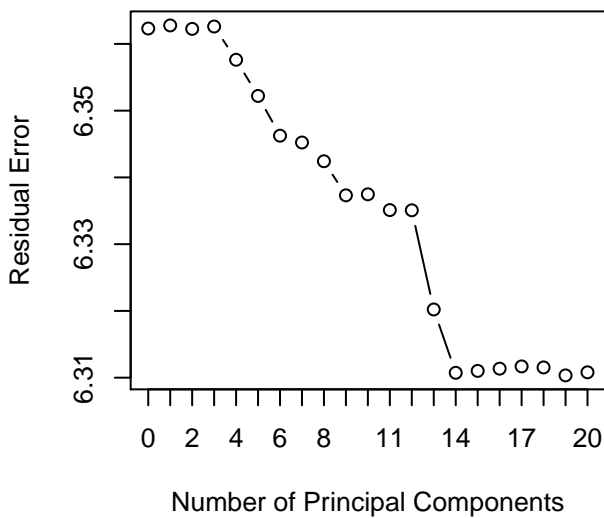

Supplement: Supplementary file 2 — Supplementary material 2 (PDF 4 kb) [file 10519_2014_9644_MOESM2_ESM.pdf]
